# An Assessment of the Phytoremediation Potential of Planted and Spontaneously Colonized Woody Plant Species on Chronosequence Fly Ash Disposal Sites in Serbia—Case Study

**DOI:** 10.3390/plants11010110

**Published:** 2021-12-30

**Authors:** Olga Kostić, Gordana Gajić, Snežana Jarić, Tanja Vukov, Marija Matić, Miroslava Mitrović, Pavle Pavlović

**Affiliations:** 1 Department of Ecology, Institute for Biological Research “Siniša Stanković”—National Institute of the Republic of Serbia, University of Belgrade, Bulevar Despota Stefana 142, 11060 Belgrade, Serbia; gugol@ibiss.bg.ac.rs (G.G.); nena2000@ibiss.bg.ac.rs (S.J.); marija.pavlovic@ibiss.bg.ac.rs (M.M.); mmit@ibiss.bg.ac.rs (M.M.); ppavle@ibiss.bg.ac.rs (P.P.); 2 Department of Evolutionary Biology, Institute for Biological Research “Siniša Stanković”—National Institute of the Republic of Serbia, University of Belgrade, Bulevar Despota Stefana 142, 11060 Belgrade, Serbia; tvukov@ibiss.bg.ac.rs

**Keywords:** fly ash, *Tamarix tetrandra*, *Robinia pseudoacacia*, *Populus alba*, *Amorpha fruticose*, bioaccumulation factor, translocation factor, trace elements phytostabilization, phytoremediation efficiency of trees

## Abstract

In this study, the potential of planted (*Tamarix tetrandra* Pall. ex M.Bieb. and *Robinia pseudoacacia* L.) and spontaneously colonized (*Amorpha fruticosa* L. and *Populus alba* L.) woody species for the phytoremediation of potentially toxic trace elements (TEs) such as As, B, Cr, Cu, Mn, Ni, Se, and Zn, from the chronosequence fly ash (FA) deposit lagoons (L1 and L2) at the ‘Nikola Tesla A’ Thermal Power Plant (TENT-A) in Serbia were analyzed. The differences in the pseodototal and bioavailable (DTPA-extractable) concentrations and mobility (AR index) of TEs in FA at the examined lagoons are a result of the time-conditioned influence of weathering (3 and 11 years respectively) and vegetation development on changing the basic physical and chemical properties of FA (texture, pH, EC, CEC, C, N, and bioavailable P and K) and its toxicity. This resulted in differences in the concentration of TEs in the roots and leaves of the examined plants at L1 and L2. All examined species accumulated Cr the most in the root (BAF > 1 and TF < 1), which suggests that they are good stabilizers of this element. Biological indices for As (BAF > 1 and TF < 1) identified *T. tetrandra* and *A.* *fruticose* as good stabilizers of As. *P. alba* stood out as accumulating the highest levels of B, Ni, and Zn, *T. tetrandra* the highest levels of Cu, Mn, and Se, and *R. pseudoacacia* the highest levels of As and B in leaves (BAF > 1; TF > 1), which makes them good extractors of these elements from the FA at TENT-A. However, due to toxic concentrations of As, B, Se, and Zn in their leaves, they are not recommended for the phytoremediation of the investigated lagoons through the process of phytostabilization. Under conditions of elevated total Cu and Ni concentration in FA, the content of these elements in the leaves of *A. fruticosa* at both lagoons were within the normal range. This, in addition to a good supply of essential Zn, the stabilization of As and Cr in the roots, an increase in BAF, and a decrease in TF for B with a decrease in its mobility in ash over time, singles this invasive species out as the best candidate for the phytostabilization of TEs in FA at the TENT-A ash deposit site.

## 1. Introduction

Humankind’s growing needs for electricity have been reflected in the increasingly intensive combustion of coal as the most important resource for its production. As a result, global coal fly ash production (FA) has increased and in 2016, reached 1.143 × 10^9^ kg per year [1]. For this reason, the last few decades have seen extensive research into finding ways to use FA in various sectors, such as the construction material industry, road building, and also soil amelioration [2]. Namely, recent research, which is in line with the European Union’s Action Plan on the Circular Economy, encourages the use of waste products like ash and municipal sewage sludge as fertilizers and macronutrient sources in sustainable plant biomass production, all aimed at reducing their disposal and the use of mineral fertilizers [3,4]. However, despite this, the utilization of such huge amounts of FA is generally very low, which is why most FA (75%) is disposed of at landfills or basins close to thermal power plants [5]. FA dispersal by wind from these disposal sites, into surrounding ecosystems, as well as the leaching or seepage of toxic substances into groundwater, represents a constant source of pollution of air, water, and soil [6,7].

The revegetation of ash deposit sites is one of the best solutions for the physical and chemical stabilization of this mobile substrate because, with their root systems, plants absorb and bind trace elements (TEs), thus preventing erosion and their leaching, while improving habitat quality both microclimatically and aesthetically [8,9,10,11]. FA is a complex, heterogeneous material, silty in texture with extremely variable physical and chemical characteristics conditioned by the geological origin of the coal, the combustion process, storage method, length of exposure to weathering (age of ash), morphological characteristics, and the size of ash particles, which have a considerable impact on its chemical composition and the solubility of chemical elements [11,12]. It is mainly composed of essential chemical elements, but also contains non-essential and toxic elements which can have a negative effect on plant growth and metabolism. In addition to Si, Al, Fe, Ca, Mg, and K, which form its major matrix (90–99%), FA contains numerous TEs, such as As, B, Cd, Cr, Cu, Co, Mn, Mo, Ni, Pb, Se, and Zn; their concentrations can be up to 30 times higher during the combustion of organic materials in coal, which makes fly ash potentially toxic for living organisms [13]. Besides TE toxicity, FA is characterized by a poor water-air ratio, extreme acidity or alkalinity, high electrical conductivity (EC) due to a high content of soluble salts, and a lack of organic matter and essential nutrients (nitrogen and phosphorous) [11]. Given the highly unfavorable physical and chemical characteristics of ash, the introduction of vegetation at these anthropogenic sites is a very challenging process, especially if undertaken directly on FA without the use of an insulating soil layer. This is why it is of the utmost importance to choose the right plants; those that are pollution tolerant and are the best at growing naturally [14,15,16,17,18,19,20]. Previous research has shown that invasive herbaceous species like *Dactylis glomerata* L., *Ipomea carnea* Jacq., *Saccharum spontaneum* L.*, Cynodon dactylon* L., and *Calamagrostis epigeyos* L. [17,21,22,23], but also invasive woody species such as *Robinia pseudoacacia* L., *Betula verrucosa* Ehrh., *Salix alba* L., *Salix fragilis* L., *Populus tremula* L., *Populus nigra* L., *Populus alba* L., *Pinus sylvestris* L., *Alnus glutinosa* (L.) Gaertn and *Alnus incana* (L.) Moench, *Paulownia* sp. have exceptional adaptive potential to survive under such extreme conditions, i.e., they do not require any specific habitat management for survival, and can play a significant role in the decontamination and amelioration of such habitats, especially during the initial stages of the revegetation process. [16,24,25,26]. In terms of FA, it has been shown that biological/technical reclamation and afforestation measures, combined with the spontaneous succession of plants, can contribute to the improvement of unfavorable physical and chemical characteristics of ash, including a reduction in the toxic concentrations of TEs in ash as the substrate on which plants grow, and may be advantageous for the development of dense and relatively species-rich vegetation on fly ash landfills and other disposal sites [27,28,29,30].

Various phytoremediation technologies that remove pollutants from polluted soils or transform them into a state that is not toxic for living beings are a cost-effective, non-invasive alternative or technologies that are complementary to engineering-based remediation methods whereby plants are used for the extraction, stabilization, degradation, or volatilization of pollutants [31]. At landfills and open cast disposal sites, plants have developed various adaptation mechanisms that allow them to tolerate nutrient deficiencies on the one hand and heavy metal/metalloid toxicity on the other, using two basic strategies based on the mechanisms of accumulation (accumulator plants) and exclusion (stabilizer plants) [32,33,34,35]. Thanks to the development of the internal mechanisms of complexation, compartmentalization, deposition, etc., accumulator plants tolerate high concentrations of TEs in their tissue, or accumulate them even in low concentrations in the substrate, without significant physiological and/or morphological damage [36]. In these plants, the content of TEs in plant roots in relation to that in the substrate (i.e., the Bioaccumulation Factor—BAF), as well as the leaf to root concentration ratio (i.e., the Translocation Factor—TF) is higher than 1 [32]. In contrast, stabilizing plants, when there are high concentrations of chemical elements in the substrate, absorb only a small part of them, i.e., their adaptation mechanisms are based on the immobilization of toxic chemical elements in the rhizosphere by the secretion of root exudates or the storing of absorbed elements in cell walls and root cell vacuoles, thanks to which translocation into the aboveground parts of the plant is low [37]. With stabilizer plants, the BAF of their roots may be greater than or less than 1, but the TF is always less than 1. The most tolerant plant species are those that absorb and translocate the smallest amounts of toxic elements into the aboveground parts in relation to their concentration in the substrate (BAF < 1; TF < 1) [9,38]. Therefore, for the remediation of extremely large and highly contaminated areas with several pollutants present, as is the case with FA disposal sites, the use of phytostabilizer plants is of greater importance [23,39]. The use of accumulator plants in such habitats would allow the cycling of undesirable elements through the litter in the substrate/plant system, which would lead to their spread through the ecosystem. Conversely, by immobilizing contaminating elements on and in the roots and in the rhizosphere, excluder plants reduce their mobility, leaching and ecotoxicity, and through the effective covering of the substrate prevent particle dispersion and erosion caused by wind and surface water runoff [40].

Determining the biologically available concentration of TEs allows us to assess the risk of their uptake and accumulation in plants, which is why the use of DTPA has been used in previous studies to understand their bioavailability in FA and their transfer to plant tissue [33,41,42]. The proportion of the biologically available fraction in the total concentration of the tested element in the substrate indicates its mobility, affected by numerous physical, chemical, and biological processes and interactions between them, such as total concentration, granulometric composition, organic matter content, sorption capacity, cation form, oxidation reduction potential, activity of microorganisms, and, most importantly, pH levels [43,44,45,46]. As far as FA is concerned, these factors have been shown to change over time, resulting in changes in the level of TEs mobility [12,27].

The majority of previous studies have most often tested the accumulation of Tes according to crop species, as well as the ability of terrestrial and aquatic herbaceous species and algae to phytoremediate FA deposit sites around the world [47,48,49,50,51,52,53]. In our previous research at the disposal site of the largest thermal power plant—‘Nikola Tesla A’ (TENT-A)—in Serbia, we identified *F. rubra*, *C. epigeyos*, and *D. glomerata* as vital elements for large-scale reclamation [17,21,41]. In contrast, a significantly smaller number of studies have evaluated the phytoremediation efficiency of woody species—the main advantage they have over herbaceous plants is reflected in their higher potential for TEs accumulation due to their large underground and aboveground biomass and rapid growth [15,54,55,56,57].

**Figure 1:**
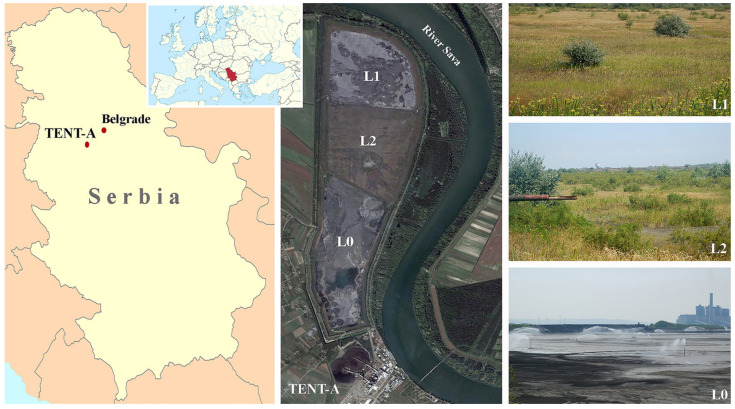
Location map showing the fly ash disposal site at the thermal power plant ‘Nikola Tesla A’ (TENT—A), Obrenovac (Serbia); L0—active lagoon, L1 and L2—passive lagoons (study sites).

In view of the above, the present study aims to determine the phytoremediation potential of four woody species, two planted (tamarix—*Tamarix tetrandra* Pall. ex M.Bieb. and black locust—*Robinia pseudoacacia* L.) and two spontaneously colonized invasive species (false indigo-bush—*Amorpha fruticosa* L. and white poplar—*Populus alba* L.), whose presence was determined at lagoons L1 and L2 (revegetated 3 and 11 years ago respectively) at the disposal site of the TENT-A (Figure 1). In our research, we started from the hypothesis that invasive woody species which spontaneously colonize the FA lagoons will be more suitable for the stabilization of the TENT A deposit site than those species that are planted as part of the revegetation process. Hence, the detailed objectives of this study were to: (1) Determine the main physical and chemical properties of chronosequence FA lagoons L1 and L2, (2) assess the different forms (pseudototal and bioavailable) of the selected TEs (As, B, Cr, Cu, Mn, Ni, Se, and Zn) in FA, (3) estimate element uptake by the examined planted and naturally growing woody species, and distribution between root and leaf portions, and (4) calculate the BAF and TF of each element for the examined species at the FA lagoons. These research findings will contribute to existing knowledge on the potential use of these species for the in situ phytoremediation of FA lagoons at TENT-A and similar habitats worldwide.

## 2. Results and Discussion

### 2.1. Physical and Chemical Characteristics of Fly Ash

Ash from lagoon L1 had a high proportion of the sand fraction and less of the clay and silt fraction, i.e., a loamy sand texture (Table 1).

This resulted in the weaker binding of ash particles and low water and nutrient retention capacity in this lagoon, which is why conditions for the establishment of vegetation and plant growth at FA deposit sites are generally very unfavorable [10,19,58]. In the ash at lagoon L2, a lower sand content and a higher clay and silt content was found compared to the ash at lagoon L1, i.e., a sandy loam texture. In addition, a higher CEC at L2 than at L1 (Table 1) meant that there was the development of capillarity in the ash L2 and an improvement in the sorption capacity and water regime. The development of woody vegetation at the older lagoon contributes to the development of aggregates and their stability which, based on the positive correlation of organic carbon, total nitrogen and underground biomass content with the formation of macroaggregates, suggested that natural shrub restoration measures reduce erosion processes significantly [59].

Ash from both lagoons is alkaline and has a low carbon and nitrogen content (Table 1). However, lower pH and salinity were observed in the ash at L2, as well as a higher N content compared to L1 as a result of the decomposition of the litter of N-fixing plants [60], such as black locust and false indigo, but also due to colonization by woody species such as white poplar, which is well adapted to moist and alkaline soils [61]. The narrower C/N ratio at L2 compared to L1 points to the faster decomposition of organic matter at the older lagoon due to more favorable conditions for intensifying the activity of microorganisms [10,62]. Namely, the development of vegetation, which is a source of nutrients and a habitat for soil fauna, contributes to the activation of biological processes in ash and the creation of more favorable conditions for further growth [63]. In addition, the accumulation of organic matter on the surface of the ash is accompanied by the colonization and increased activity of fungi and bacteria, which decompose cellulose [64]. It has previously been determined that the development of microorganism populations occurs on revitalized and unrevitalized post-mining sites, but the presence of vegetation significantly accelerates this process [65]. After 17–20 years, lignite ash at a deposit site in the city area of Halle, Saxony-Anhalt in Germany was colonized by populations of microorganisms tolerant to conditions that predominate in ash, but are significantly different from populations in the surrounding soil [66]; another study, which followed the accumulation of organic matter and microbial activity for 25 years at the tailings ponds of the Pernik mine in Bulgaria, revealed that afforestation with black locust had a positive impact on the fast transformation of plant litter and the formation of mobile organic matter, which migrates into the mineral profile, and the beginning of the soil formation process [67]. Namely, the significant impact of nitrogen-fixing plant species is reflected in the fact that, as early colonizers of such habitats, they are tolerant to high levels of boron salts and heavy metals [68] and that by taking up atmospheric N, they enrich the substrate through fast decomposing litter [60]. The enhancement in the buffering capacity of FA through litter decomposition, the exchange of ions during nutrient uptake by plants, and also an increase in the root zone content of CO_2_, whose dissolution in water produces weak carbonic acids [69], causes a lower pH of the ash at L2. An increase in the content of available potassium (K_2_O) in FA during vegetation development at the TENT-A ash deposit site (Table 1), but also of available phosphorus (P_2_O_5_) had also been observed in other studies [27,62]. Nevertheless, the addition of the NPK fertilizer at the beginning of the revegetation process brought about a medium level of available phosphorus at L1, while at L2 its content was low, which may be the result of its leaching from the aluminosilicate ash matrix due to precipitation and a decrease in pH over time [70]. In order to ensure improvements in fertilization techniques in changeable environmental conditions and the ever-greater presence of pollution from heavy metals and other toxic substances, which result in changes in biological activity and the soil nutrient pool, research has been conducted in the impact of different kinds of fertilizer on soil microbial and enzymatic activity [71]. It confirms the superiority of using complex mineral and organic (green manure and farmyard manure) fertilizers, as well as introducing new fertilization techniques using nanofertilizers, the particles of which release nutrients to plant cells more efficiently. The application of such fertilizers results in an increase in enzymatic activity as indicators of microbial activity in soil, which is of particular biological importance in the decomposition of plant/animal remains, the release or binding of TEs and nutrients, maintaining soil fertility, and creating favorable conditions for plant growth [71,72]. In general, the results showed that the physical and chemical characteristics of FA at L2 are more favorable for plant growth than those at L1. A similar trend in terms of an improvement in the physical and chemical characteristics of ash as it aged and as the revegetation process progressed was observed in previous research at the TENT-A deposit site [17,27], as well as at other ash deposit sites around the world [62,73,74].

### 2.2. The Concentration of Examined Trace Elements in Fly Ash and Their Bioavailability

The pseudototal concentration (C_PT_) of TEs (As, B, Cr, Cu, Mn, Ni, Se, and Zn) in the surface layer (0–30 cm) of FA at lagoons L1 and L2, of which B, Cu, Mn, Ni, Se, and Zn are essential, and As and Cr are non-essential micronutrients [75,76], fell within the range of typical values for different types of ash (Table 2 [11]).

At L1, the concentration of As and Cr in the ash at this lagoon was in the critical range for plants (Table 2 [77]), while the B, Cu, Ni, and Se concentration was higher than the average values, the Zn concentration fell within the range of average values, and Mn was lower than the average values for sandy to silty loam soils (Table 2 [45]). At L2, C_PT_ of Ni and Se was similar to the concentration at L1, while the As, B, Cr, Cu, Mn, and Zn concentration was lower than in the ash at L1, as a result of weathering and the revegetation process [17,23,34]. Our previous research into the effects of weathering and vegetation on the development of substrate properties at L1 and L2 (3 and 11 years after revegetation) revealed a reduction in total concentrations of As, B, Cr, Cu, Mn, and Zn over time. Namely, changes in concentrations of these TEs were determined from fly ash via active lagoon (L0–initial bare ash) to inactive lagoons L1 and L2, with the greatest reduction in their concentrations coming during the hydraulic transport of the ash to L0 (62%, 91%, 52%, 40%, 12%, and 40% respectively) and at L1 compared to L0 after 3 years (64%, 57%, 50%, 42%, 35%, and 39% respectively). This is explained by the dominant influence of weathering due to the poor development of the vegetative cover, above all trees. However, the reduction in TEs at L2 compared to L1 was less intense (13%, 28%, 22%, 16%, 2%, and 10% respectively) due to a reduction in leaching and the greater impact of vegetation, which had developed at L2 over the 11-year period [27]. More precisely, C_PT_ of As, Cu, Ni, and Se in ash at L2 was higher than the average values, B fell within the range of average values, and Cr, Mn, and Zn was lower than the average values for sandy to silty loam soils (Table 2 [45]). C_DTPA_ of TEs in FA were found to be relatively low, with the following order at L1: B > Mn > Cu > Zn > Ni > As > Cr > Se, and at L2: Ni > Mn > As > Cu > Zn > B > Cr > Se. A higher C_DTPA_ of B, Cr, Cu, Mn, and Zn was determined in the ash at L1, and of As and Ni in the ash at L2, while the Se concentration was similar at both lagoons (Table 2). A similar bioavailable concentration of the individual elements was determined in previous research, with the following order: Zn (1.2 mg kg^−1^) > Cu (0.9 mg kg^−1^) > Ni (0.56 mg kg ^−1^) > Mn (0.4 mg kg ^−1^) [53], and Cu (1.4 mg kg^−1^) > Mn (1.2 mg kg ^−1^) > Zn (0.95 mg kg^−1^) > Ni (0.3 mg kg^−1^) > Cr (< detection limit) [33]. Based on the obtained results, the availability ratio index (AR) was also calculated. For B, Cu, Mn, and Se, it was higher in the ash from L1, while for As, Cr, Ni, and Zn, it was higher in the ash from L2, i.e., for all the examined elements apart from Cr and Zn, changes in AR followed the trend of changes in the C_DTPA_ concentration. This can be explained by greater differences in C_PT_ of Cr and Ni compared to the differences in the C_DTPA_ of these two elements in the FA from the investigated lagoons. Based on this index, the mobility of the examined elements in the ash decreased in the following order at L1: Se > B > Cu > As > Ni > Zn > Mn > Cr, and at L2: As > Se > Cu > Zn > B > Ni > Mn > Cr (Table 2). Namely, the ash from TENT-A, just like all alkaline ashes, is characterized by a higher content of mobile As, B and Se [58], which can have a very unfavorable effect on plants growing at L1 and L2, since these elements are very easily accessible to plants and plants accumulate them in their tissue [17,21,78].

The decreasing pH of the FA over time should result in an increase in the affinity of As to form oxides with Fe, Al, and Mn, and a reduction in its solubility [79]. However, the complex leaching behavior of As is conditioned by salt content, especially Fe (II) sulfate, the higher content of which at L1 effectively reduces its mobility at this lagoon, and by the content of organic matter, whose higher content at L2 reduces As (V) to the much more toxic and more mobile As (III) [80,81]. Namely, the higher content of fulvic acids found in the ash from L2 relative to L1, as the best indicator of the higher organic matter content in ash at L2 [82], could displace As from organic/inorganic binding sites and increase its mobility [83]. Decreasing pH also increases the solubility of B [84]. However, at pH > 6 its mobility is conditioned only by its total concentration [85], due to which the bioavailability of B is higher at L1. The solubility of Se in water in alkaline ash is very high and can reach up to 50% [85]. It is highest at pH 10–12 and lowest when the pH is close to neutral [44]. Therefore, most Se is leached from the ash at TENT-A during its hydraulic transport, so differences in C_PT_ of Se as well as its C_DTPA_ concentration at L1 and L2 were not determined. A decrease in pH in the presence of organic matter stimulates the reduction of easily soluble Cr^−6^ to poorly soluble and less toxic Cr^−3^ [86,87], which could be the cause of the lower C_DTPA_ of Cr at L2. The association of Cu in primary crystals in FA at the TENT-A deposit site [88], chemisorption in the presence of carbonates, phosphates and clay minerals, and the pronounced affinity of Cu for binding to organic matter [45,89] are the causes of its lower bioavailable content at L2. This is in contrast to previous studies which found an increase in bioavailable Cu from 0.26 to 0.28 mg kg^−1^ in ash, three years after establishment of the plantation [8]. At lagoons L1 and L2, the ash is well drained and the pH is neutral to slightly alkaline, which makes Mn less mobile [90], and the differences in its C_DTPA_ concentrations are a reflection of the differences in total concentrations at the different-aged lagoons. Ni solubility is lowest at pH 8–10 and increases with increasing acidity [44]. Furthermore, the higher content of organic matter at L2 mobilizes Ni from carbonates and oxides and reduces Ni sorption on clay particles, forming organic ligands in which Ni is not so tightly bound [45], which increases the bioavailability of Ni at this lagoon. The lower bioavailable Zn concentration in the ash from L2 is the result of a continuous decrease in C_PT_ of Zn in the ash during its disposal, as shown by our previous research [17,27].

### 2.3. The Accumulation of Examined Trace Elements in the Roots and Leaves of Four Woody Plant Species

Under the same conditions, different plant species absorb different amounts of chemical elements, which can then be transported, converted, stored, and accumulated by different tissues and cells [91].

In addition to their concentration, bioavailability, and mutual interaction, their uptake is influenced by a multitude of physical and chemical factors related to the substrate (texture, humidity, pH, absorption capacity, organic matter content, etc.), but also plant age, species variety, vegetation period, etc. [75,78]. Therefore, determining the concentration and mobility of TEs in the tissues of plants growing at ash deposit sites is important so as to determine their specific behavior and phytoremediation potential in order to prevent pollution of the surrounding habitats.

**Figure 2:**
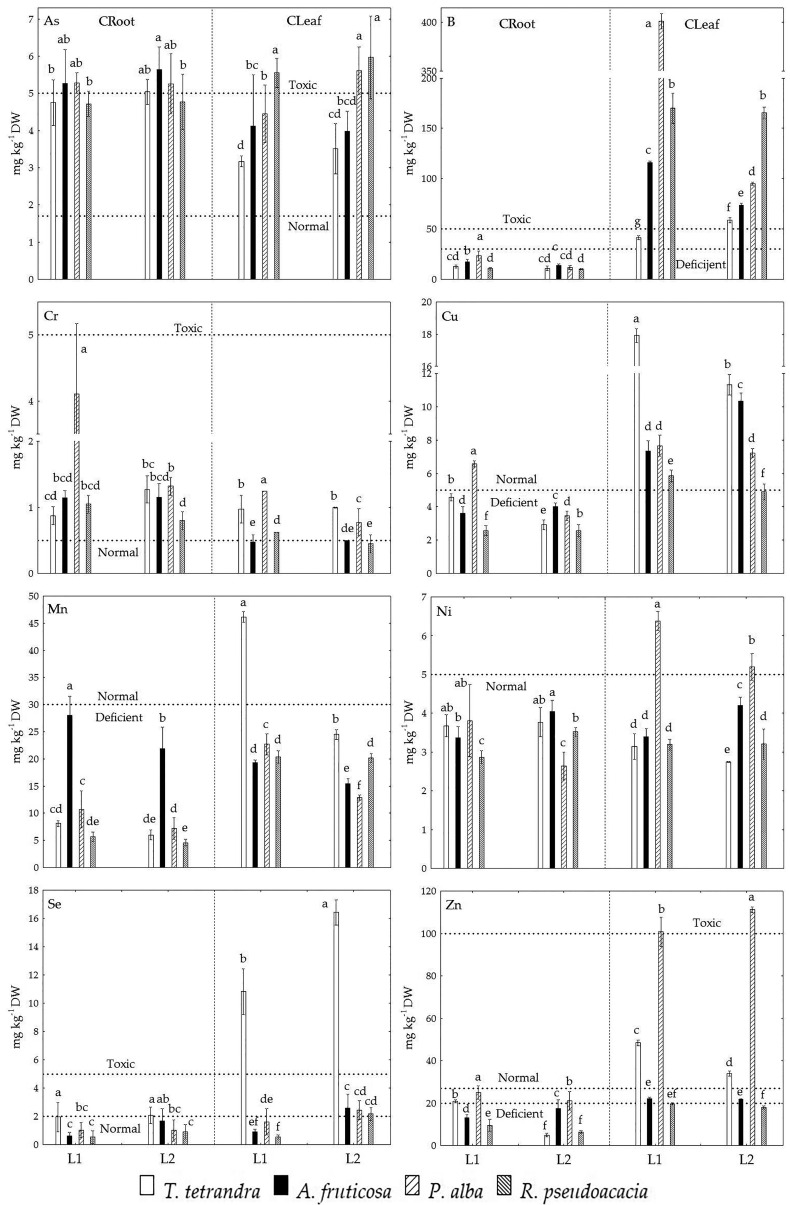
Total concentrations of trace elements in the roots (CRoot) and leaves (CLeaf) of four woody plant species growing at the examined sites (L1 and L2). DW—dry weight. Deficient concentration [45], as well as normal and toxic concentrations [45,77].

In this study, the analysis of potentially toxic but essential elements (B, Cu, Mn, Ni Se, and Zn, Figure 2, Table S1) showed that, in their leaves, the examined woody plant species from the TENT-A ash deposit site generally accumulated toxic levels of B and Se and normal concentrations of Cu, Ni, and Zn, while Mn was deficient. A deficit of essential TEs except Se was found in the roots of all the examined species apart from P. alba, for which Cu at L1 and Zn at both lagoons fell within the normal range (Figure 2). The highest B concentration was measured in white poplar leaves at L1, while in the leaves of black locust it was similar at both lagoons (Figure 2); in false indigo and white poplar it was lower at L2, where its lower mobility in ash was determined (r = 0.998, *p* < 0.001; Table 3), while in tamarix, it had the opposite trend, i.e., it was higher in individuals from L2 (r = −0.993, *p* < 0.001; Table 3).

When it comes to poplar, it has a high capacity for the uptake and accumulation of B in its leaves, and on the substrate containing 30 mg kg^−1^ of boron, poplar leaves had an average B concentration of 845 mg kg^−1^ wiyh levels aligned linearly with leaf age [92]. Furthermore, at concentrations of B of up to 900 mg kg^−1^ in the leaves of the hypertolerant species P. nigra × euramericana, less than 10% of the leaf area showed signs of toxicity [93]. It has also been determined that increased substrate salinity can reduce the accumulation of toxic B concentration in the leaves and stem of Prunus sp. and Sorghum bicolor L., which is a result of their antagonism during uptake by plants [94,95]. This feature is only characteristic of plants such as tamarisk, which is adapted to increased salinity [96,97]. The results of this study showed that the leaching of salt from the ash over time has allowed tamarix to absorb B more intensely to toxic concentrations at L2, with a tendency to increase further with decreasing substrate salinity. The low concentration of bioavailable Cu in FA, despite a higher-than-average total concentration, caused concentrations of this element in the examined plants to range from 2.570 mg kg^−1^ to 17.908 mg kg^−1^ and to be deficient in their roots while falling within the normal range in their leaves (Figure 2 [45]). Specifically, Cu in FA is most present in the form of CuO (up to 51% [98]), which plants most easily absorb. This is why they can secure a sufficient amount of Cu to allow metabolic processes to take place unhindered, despite the low content of biologically-available copper. Similar behavior of plants at ash deposit sites has been found in previous studies [14,19]. At the same time, a lower Cu concentration in plant tissues was found for all the examined species at L2, as a result of its lower mobility at this lagoon. The exception was *A. fruticosa*, which was confirmed by the negative correlation between C_DTPA_ and CRoot (Leaf) for Cu in this species (Table 3). This shows the adaptive capacity of *A. fruticosa*, as well as its development of a mechanism to provide plant tissues with a sufficient amount of essential Cu for metabolic processes to take place unhindered in conditions where there is a small amount of available Cu in FA, as confirmed by the high photosynthetic efficiency of this species in relation to the other three woody species from the same habitat [99]. The low total and bioavailable Mn concentration in the ash at L1 and L2 caused a deficiency of this element in the leaves of all the examined plants, apart from tamarix at L1 (Figure 2). Moreover, for all the species except black locust, the Mn concentration in leaves was lower at L2, where the mobility of this element in FA was lower (Table 2 and Table 3 and Figure 2). Specifically, the high availability of Fe in ash, thanks to the antagonistic behavior of Fe and Mn, can result in lower concentrations of Mn in plant tissues [45], meaning that a deficit of this element is characteristic of plants growing at ash deposit sites [17,19,53,55].

The alkalinity of FA caused Ni mobility to be generally low, because the formation of hydroxides, in which Ni is less soluble, occurs even at pH > 6.5 [100]. Therefore, concentrations of this element in the examined plants fell within the normal range, except in white poplar leaves at both lagoons, where Ni concentration was higher than normal but not toxic (Figure 2), and with a tendency to decrease from L1 to L2 despite the greater mobility of Ni at the older lagoon (r = −0.988, *p* < 0.001; Table 3). Toxic Se concentrations were only determined in the leaves of *T. tetrandra* at both lagoons, while in the roots of this species at L2 concentrations were higher than the average values (Figure 2). This may be due to the low available concentrations of N, P, Mn, Cu, and Zn in the ash from TENT-A, as shown by the negative correlation between Cu, Mn, and Zn content in the roots and Se content in the leaves of tamarisk (Table 4). Namely, the higher absorption of Se by plants is caused by the reduced uptake of Mn, Cu, and Zn, while the application of P and N contributes to the detoxification of Se, i.e., its reduced uptake by plants [45]. The Zn concentration in the roots of the examined plants at both lagoons was in the deficient range, except in white poplar, and with a tendency to decrease further at L2, except in A. fruticosa. It was previously found that Zn deficiency increases the boron permeability of the plasma membrane of root cells, which may result in the accumulation of toxic concentrations of this element in plant tissues [101]. CLeaf for Zn fell within the normal range (Table 3 [77]) for both tamarix and false indigo. The highest values of Zn, in the toxic range, were measured in the leaves of P. alba, which, can be used as a biomonitor of soil zinc pollution due to its ability to take up this element [102,103]. At the same time, the Zn deficiency found only in black locust at both lagoons may cause a lower efficiency of water uptake, nodulation, and nitrogen fixation [56,104].

In the case of examined non-essential TEs, toxic concentrations of As were found in the examined plants, as well as increased levels of Cr (Table S1). Arsenic concentration in the roots of the examined plants was fairly uniform at both lagoons, amounting to about 5 mg kg^−1^, i.e., it was bordering on being toxic, while in leaves it was higher than normal, and in white poplar at L2 and black locust at L1 and L2 it fell within the toxic range (Figure 2 [45]). This is a result of its high total content and greater mobility, but also a lack of P, especially in FA at L2 [80,105]. However, the greater mobility of As in FA from L2 did not result in higher concentrations in the leaves of the examined plants, apart from P. alba, which is also shown by the positive correlation of C_DTPA_ and CLeaf for As (Table 3). A similar pattern, i.e., a significant increase in TF with an increase in As levels in soil, was also observed for P. deltoides [106]. This can be a result of the antagonistic effect between Se and As uptake, with both selenite and selenate effective in decreasing the translocation of inorganic arsenic from the roots to their above-ground parts, which has been determined for rice [107]. In our research, the antagonistic effect between Se and As uptake was most marked in tamarisk and black locust. In the examined plants, the content of absorbed Cr in roots and leaves ranged from 0.450–4.109 mg kg^−1^, with it only being in the normal range for leaves in false indigo at both lagoons and black locust at L2 (Figure 2 [45]). The highest Cr concentration, bordering on toxicity, was found in the roots of white poplar at L1, where C_PT_ of Cr in FA was in the critical range for plants, and C_DTPA_ of Cr was higher than in the ash from L2 (Table 2 and Figure 2).

### 2.4. An Assessment of the Phytoremediation Potential of the Examined Woody Plant Species Based on Biological Indices

**Figure 3:**
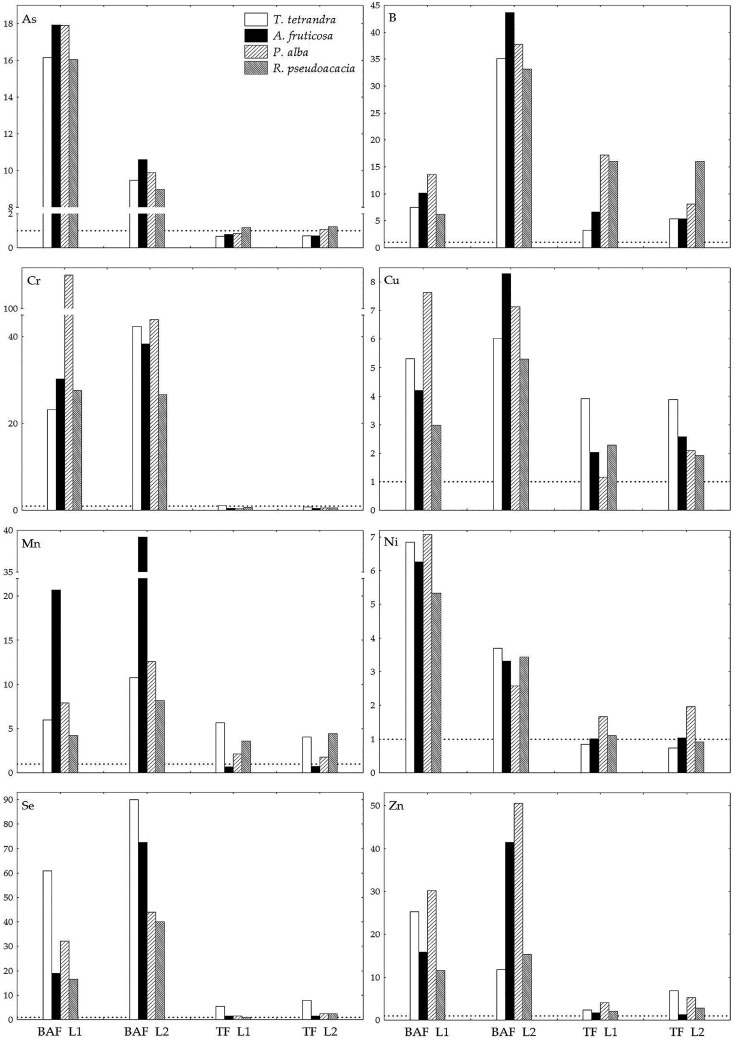
The Bioaccumulation (BAF) and translocation factor (TF) of trace elements in four woody plant species growing at the examined sites (L1 and L2).

**Figure 4:**
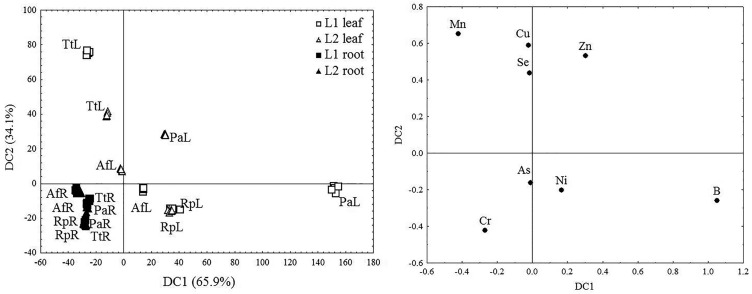
Canonical discriminant analysis (CDA) based on trace element concentrations in the roots ^®^ and leaves (L) of the examined species (Tt—*T. tetrandra*, Rp—*R. pseudoacacia*, Pa—*P. alba*, and Af—*A. fruticosa*) from fly ash disposal sites L1 (squares) and L2 (triangles).

In order to evaluate the potential of the selected woody plants for the phytoextraction and phytostabilization of TEs, the bioaccumulation (BAF) and translocation (TF) factors were calculated (Figure 3, Table S2), while species were classified according to their accumulation ability on the basis of canonical discriminant analysis (CDA) results (Figure 4).

It was found that all the examined plants at L1 and L2 accumulate the studied TEs in their roots (BAF > 1), while the translocation factor values 1 < TF > 1 (Figure 3). Based on the first component (DC1), which explains 65.9% of the differences, P. alba stood out as having the highest accumulation of B, Ni, and Zn in its leaves, while Cr accumulated most in the roots of all the examined species. Based on the second component (DC2), which explains 28.6% of the differences, *T. tetrandra* stood out for having the highest accumulation of Cu, Mn, and Se, while *R. pseudoacacia* had the highest accumulation of As and B in its leaves (Figure 4).

The results of the research showed that the examined plants accumulated essential elements in their tissue (BAF > 1, TF > 1; Figure 3). The highest translocation of B was found in white poplar at L1 (Figure 3 and Figure 4). Thanks to strong transpiration, poplar leaves can accumulate from 500–1200 mg kg^−1^ of B, which is why it is often used in the processes of phytoextraction and the reduction of B leaching from contaminated substrates into groundwater [78,92].

In our study, a decrease in TF for B with a decrease in the amount of bioavailable B was observed in false indigo and white poplar, while in tamarisk, TF exhibited the opposite trend (Figure 3; Table 4). This indicates that false indigo has developed adaptive survival mechanisms under conditions of elevated B concentrations, while the lower B concentration when compared to white poplar and black locust makes it more suitable for the phytoremediation of habitats with elevated B levels. The increase in TF in tamarix may indicate that the long-term exposure of this species to stress has led to a decrease in its tolerance to B. Namely, it is considered that more tolerant plant species, as well as more tolerant varieties within the same plant species, have a lower B concentration in their vegetative organs [108], i.e., due to the reduced permeability of membrane lipids and/or the reduced presence of B carriers (BOR and NIP), they exclude B from the cytoplasm, i.e., exclude it at root level [109,110].

The pattern of metal accumulation varies significantly for different plants, so the greater accumulation of Cu in the leaves of plants at the TENT-A ash deposit site in relation to Cu accumulation in the roots is in accordance with the results of Rawat et al. [111], but contrary to the results of Maiti and Jaiswal [33] and Tripathi et al. [112]. The higher bioaccumulation and translocation of Cu in false indigo at L2 compared to L1, together with sufficient and normal concentrations of this element in the leaves, sets this species apart from others (Figure 2 and Figure 3) and shows that in conditions of lower bioavailable Cu concentration in the FA at the older lagoon (Table 2; Table 3), this species has developed a mechanism that allows it to best provide plant tissue with this essential microelement. Since Mn and Zn are essential microelements, plants easily absorb them and transport them to the leaves [45,77]. This was determined in our research by TF > 1, for all the examined species except for Mn in false indigo (TF < 1), where the deficit of this element in the leaves was the highest (Figure 2 and Figure 3). The translocation of Mn in all plants which spontaneously inhabit fly ash lagoons in India was also determined by Mukhopadhyay et al. [14]. On the other hand, the Mn deficiency in the roots of false indigo was the lowest of all the examined species, which may provide it with greater resistance, since Mn deficiency leads to a decrease in the lignin concentration in plant tissue, especially in the roots [113]. At both lagoons, all the species accumulated Se in their roots (BAF > 1) and transported it to their leaves (TF > 1), with the highest TF found in tamarisk (Figure 3), which sets it apart from the examined plants as the most important extractor of this element from the ash at TENT-A (Figure 4). Plants easily absorb soluble forms of Se in the form of selenite (SeO3^2−^) and even more in the form of selenate (SeO4^2−^) [114]. In alkaline conditions, such as those at L1 and L2, selenate predominates in ash and it is characterized by faster distribution through plant tissue, where TF can range from 1.4–17.2 [115]. Moreover, selenate had a stronger inhibitory effect on the translocation of As from roots to leaves than selenite [107], as shown by the lowest As content in the leaves of tamarisk, which had the highest Se concentration of all the examined species at both lagoons. The uptake of Ni by plants is conditioned by the concentration of Ni^2+^ cations in the substrate, plant metabolism, acidity of the soil solution, the presence of other metals, and the composition of organic matter [116]. The main factors that cause Ni deficiency are: An excessive amount of Cu^2+^ and Zn^2+^, which competitively inhibit the uptake of Ni, pH > 6.5, which causes the formation of poorly soluble Ni hydroxides and oxides, a high concentration of N, Fe, Mn, Ca, or Mg in the substrate, and high phosphorus content, which favors the formation of Ni phosphate and reduces Ni absorption by plants [99]. Nevertheless, the low content of competing metals in ash could not inhibit either the uptake of Ni from FA, so BAF > 1 was found for all the examined species, or the increased transport of Ni from the roots to the leaves, which was most pronounced in white poplar (TF ≈ 2), (Figure 3). The most marked stabilization of Ni was found in tamarix (TF < 1), but also in false indigo and black locust, in which TF ≈ 1 and Ni concentrations fell within the normal range even though the total concentration of this element in FA at both lagoons was elevated (Table 2, Figure 2 and Figure 3).

In conditions of elevated As levels in ash, all the examined species accumulated this non-essential element in their root tissue (BAF > 1; Figure 3), but in tamarix and false indigo, the efficient mechanisms for binding the largest amount of As in roots prevented the transport of toxic concentrations of As into the leaves (TF < 1; Figure 3). This may indicate one of the tolerance mechanisms of these two species based on the principle of the exclusion of As at the root level (excluder plants), and the potential of these two species to stabilize a substrate with elevated As concentrations. Namely, the rapid reduction of arsenate (AsV) to arsenite (AsIII), followed by its complexation with thiols, and their further sequestration in the root vacuoles reduces As efflux to distant tissue like leaves [117,118]. Plants also absorb less toxic organic forms of As (e.g., monomethylarsonic acid and dimethyl-As acid), which are rapidly translocated through the xylem to aboveground parts [80]. In white poplar at L2 and black locust at L1 and L2, TF > 1 indicates that these two species transports most of the absorbed As to older leaves, i.e., they have As extraction potential (Figure 3), which may be their tolerance mechanism and a form of their releasing toxic levels of this element through autumn deposition [119]. At both lagoons, the examined plants used a strategy of excluding Cr at the root level (BAF > 1; TF < 1; Figure 3), except for tamarix (TF > 1) at L1, where critical Cr concentrations were found in the ash (Figure 2 and Figure 3). Compared to other trace elements, Cr is the least mobile element, meaning the highest Cr content accumulates in the roots, and the lowest in the vegetative and reproductive organs [120]. Results of research by Mukhopadhyay et al. [14] also confirm this behavior of plants towards Cr. Although all species behaved as excluders (TF < 1), the Cr content in the leaves of false indigo and black locust, which fell within the normal range, makes these two species more suitable for the phytostabilization of sites with critical and elevated Cr concentration in the substrate than tamarix and white poplar, in which the Cr concentration measured in the leaves was higher than the average values.

## 3. Materials and Methods

### 3.1. Study Sites Description

The TENT-A ash disposal site, with a total area of 400 ha, is located on the right bank of the Sava River, 41 km upstream from Belgrade, the capital of Serbia, in the municipality of Obrenovac (lat. 44°30′ N, long. 19°58′ E, average altitude 80 m), which is characterized by a temperate continental climate, with an average annual temperature of 12.5 °C and mean annual precipitation of 690.1 mm (Figure 1). It is divided into three lagoons of an approximately equal area: An active lagoon L0 (the concentrations of the studied TEs in FA from L0 are given in [27]), which the ash, combined with water (ash to water ratio 1:10), is piped into, and two inactive lagoons L1 and L2, on which the process of revegetation was carried out. After sowing the grass-legume mixture directly on the ash (composition of the mixture and sowing density are given in the research of Kostić et al. [27]), cuttings of woody species *T. tetrandra* and *R. pseudoacacia* were planted using agrotechnical measures (800 kg·ha^−1^ of mineral fertilizer 15N:15P:15K and wetting of sown areas until plant cover formation; [121]), so that the process of revegetation and aging of ash lasted 3 and 11 years (L1 and L2 respectively) at the time of the research. An analysis of the vegetative cover at L1 and L2 revealed 99 plant species (61 at L1 and 86 at L2), grouped into 32 families, of which 91 species had spontaneously colonized the deposit site (55 at L1 and 80 oat L2). Woody species account for 9% of the total number of species present (4 at L1 and 9 at L2).

**Figure 5:**
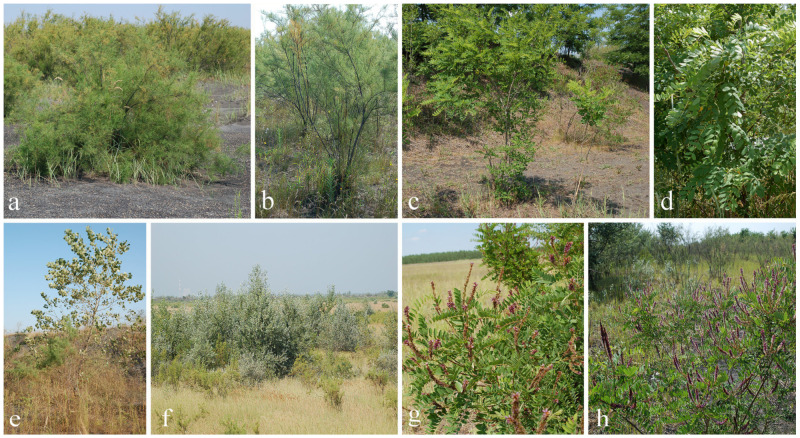
Examined woody plant species: (**a**)—*T. tetrandra* L1, (**b**)—*T. tetrandra* L2, (**c**)—*R. pseudoacacia* L1, (**d**)—*R. pseudoacacia* L2, (**e**)—*P. alba* L1, (**f**)—*P. alba* L2, (**g**)—*A. fruticose L1*, and (**h**)—*A. fruticosa* L2.

To investigate the phytoremediation potential, woody species common to both lagoons were selected (Figure 5), specifically: Planted *T. tetrandra* and *R. pseudoacacia* and the spontaneously colonized *A. fruticosa* and *P. alba*, which occur sporadically at L1, while covering 30% of the surface of L2 through natural regeneration and by colonizing the lagoon from the periphery towards the center [27].

### 3.2. Plant Species Description

*Tamarix tetrandra* Pall. ex M.Bieb. (Tamaricaceae) or tamarix is a xerophilous and heliophilous shrub or tree native to south eastern Europe, Turkey, Bulgaria, and Crimea, which, as with all species from this family, can be used for the revegetation of degraded areas prone to salinity and drought due to its rapid growth, easy vegetative propagation, and tolerance to very high salt levels (up to 15,000 ppm) in the soil solution [122]. *Robinia pseudoacacia* L. (black locust) and *Amorpha fruticosa* L. (false indigo-bush) are invasive species from the family Fabaceae, native to North America, from where they were brought to Europe in the 16th and 17th centuries, where they are widespread today. Thanks to the rapid germination of seeds and high sprouting ability, nitrogen fixing ability, and high tolerance to different ecological conditions, these species do well on different soil types, from wetlands beside river banks to dry embankments and road and railway cuttings, and with a branched root system they afford soil excellent protection against erosion. Black locust has been used widely in the afforestation and amelioration of torrent, eroded, deforested, sandy terrains, and degraded areas, such as mine tailings and ash deposit sites [123], while false indigo, which is treated as a highly noxious invasive species in Europe, can serve as a cheap resource, which can be utilized for remedial purposes [124]. *Populus alba* L. (Salicaceae) or white poplar is a native tree in the riparian steppe and coastal forests of central and southern Europe. It is a fast-growing species characterized by rapid vegetative spread and a good capacity to adapt to stress, which is why it is used to control erosion on river banks and roads, in windbreaks and land remediation [125].

### 3.3. Sample Collection and Preparation

The criteria for the selection of sampling plots (six at each lagoon, with an area of 15 × 15 m) were the presence of all four woody species, approximately equal distance from the perimeter embankment of the lagoon (25–30 m), and an even distribution along the perimeter of the lagoon at intervals of approximately 200 m. At each sampling plot, a stainless-steel shovel was used to collect approximately 250 g of ash per sample, from a depth of 0–30 cm (rooting zone), which, after drying at room temperature, was sieved through a 2.0-mm sieve, and then coned and quartered to form representative samples (~500 g) for each lagoon. Leaf samples were collected using random selection, from equal heights and all four exposures, and combined into a pooled sample for each of the examined species from L1 and L2. After washing with tap water and distilled water, the plant samples were dried to a constant weight at 65 °C and ground in a laboratory mill (Polymix, Kinematica AG, Switzerland, stainless-steel sieve 2.0 mm).

### 3.4. Physical and Chemical Analysis of Fly Ash

In the representative FA samples, the following were analyzed: Particle size distribution using combined pipette and sieve techniques with a 0.4-N solution of sodium pyrophosphate, soluble salt content by assessing electrical conductivity (EC dS/m) in a 1:5 fly ash to water (distilled) suspension (Knick, Germany, Portamess 911 Conductometer), and pH values, active (pH_H2O_) and substitutional (pH_KCl_), in a water and KCl suspension (fly ash: solution ratio of 1:5 *w*/*v*) (WTW-Germany, inoLab 7110 pH meter). In addition, total organic carbon content (C%) was analyzed through titration, using (NH_4_)2Fe (SO_4_) 2 × 6H_2_O after the digestion of samples with a dichromate-sulphuric acid solution, based on Simakov’s modification of the Turin method [126], as well as total nitrogen content (N%), using the semimicro-Kjeldahl method, and their ratio (C/N) was calculated. Available phosphorus (P_2_O_5_ mg/100 g) and potassium (K_2_O mg/100 g) were extracted with ammonium acetate-lactate (AL solution, pH 3.7, ratio 1:20) and determined by flame photometry [127]. Cation-exchange capacity (CEC cmol/kg) was determined using the Kappen method [128]. All physical and chemical analyses were performed in 3 replicates (*n* = 3).

### 3.5. Pseudototal and Bioavailable Concentration of Trace Elements in Fly Ash

To analyze the pseudototal concentration (C_PT_) of TEs (As, B, Cr, Cu, Mn, Ni, Se, and Zn), FA samples (0.5 g), passed through a 0.2-mm diameter sieve, were mineralized in an aqua regia mixture (3 mL of 65% HNO_3_ and 9 mL of 37% HCl), using a microwave oven (CEM, Mars 6 Microwave Acceleration Reaction System, Matthews, NC, USA). After filtration, the digestion products were adjusted to a volume of 50 mL with deionized water. To assess the bioavailable pool of TEs, FA samples were subjected to extraction with DTPA (C_DTPA_) (10 g of FA with 20 mL of 0.005 M diethylene-triamine-pentaacetic acid-DTPA, 0.01 M calcium chloride dihydrate-CaCl_2_·2H_2_O, and 0.1 M triethanolamine-TEA, with the pH of the solution adjusted to 7.3) [129]. Analyses of pseudototal and bioavailable concentration of TEs were performed in 5 replicates (*n* = 5).

The concentration of TEs (mg kg^−1^) in FA obtained after these extractions was determined by inductively-coupled plasma optic emission spectrometry (ICP-OES, Spectro Genesis, Spectro-Analytical Instruments GmbH, Kleve, Germany). The detection limits for the analyzed elements were as follows (mg kg^−1^): As—0.005, B—0.005, Cr—0.001, Cu—0.001, Mn—0.001, Ni—0.009, Se—0.007, and Zn—0.005. The accuracy of the analytical procedure was confirmed by analyzing certified reference material (ash from coal BCR—038 and sediment certified reference material BCR—701 for three-step sequential extraction; IRMM, Institute for Reference Materials and Measurements, Geel, Belgium; certified by EC-JRC, European Commission—Joint Research Centre). The average recovery values for elements in the standard reference materials were in the range of 100 ± 20%.

### 3.6. Estimation of Trace Elements in Plants

The digestion of plant material samples (0.4 g) was also performed in a microwave oven (CEM, Mars 6 Microwave Acceleration Reaction System, Matthews, NC, USA), in 5 replicates (*n* = 5), in a mixture of 9 mL of 65% HNO_3_ and 3 mL of 30% H_2_O_2_ [130]. After filtration, the digestion products were made up to a volume of 50 mL with deionized water and concentrations (mg kg^−1^) in plant tissues were measured by inductively-coupled plasma optic emission spectrometry (ICP-OES, Spectro Genesis, Spectro-Analytical Instruments GmbH, Kleve, Germany). The accuracy of the measured values was confirmed by analyzing standard reference material (Beach leaves—BCR-100, IRMM certified by EC-JRC), with recovery values in the range of 100 ± 15%.

### 3.7. Chemicals

All chemicals used in this research were of analytical grade (Merck, Darmstadt, Germany). The ICP-multi-element standard stock solutions (concentration of elements, 1000 mg L^−1^ in diluted nitric acid) used to prepare standard solutions for ICP-OES analysis were also obtained from Merck.

### 3.8. Statistical Analysis

All values in Table 1 and Table 2 and Figure 2 are presented as the mean (M) with the standard deviation in parentheses (SD). The data from this study was analyzed using statistical analysis (ANOVA) and means were separated with a Bonferroni test at a level of significance of *p* < 0.05, using the Statistica software package (StatSoft In., Tulsa, OK, USA, 2007). It was checked that data met the assumptions for ANOVA prior to it being analyzed. The mobility of TEs in FA was assessed using the availability ratio index (AR), calculated according to Formula 1 [43]:

AR (%) = C_PT_ × 100/C_DTPA_. (1)

Correlations between the levels of the examined elements in FA and root and leaf samples were obtained using the non-parametric Spearman rank-order correlation at a level of significance of *p* < 0.05, *p* < 0.01 and *p* < 0.001 (Table 3 and Table 4). The efficiency of plants to bind or remove chemical elements from the substrate and transport them from the roots to the leaves was compared by assessing biological indices such as the bioaccumulation factor (BAF) and the translocation factor (TF), which were calculated according to Formulas (2) and (3) respectively [131]:

BAF = CRoot/C_DTPA_ (2)

TF = CLeaf/CRoot.(3)

CRoot and Cleaf represent the concentrations of the selected element in the roots and leaves, respectively. Classification of species based on their ability to accumulate TEs in their roots and leaves was performed on the basis of the results of canonical discriminant analysis (CDA) (Figure 4).

## 4. Conclusions

This study showed that, besides the impact of different basic physical and chemical characteristics and pseudo total and bioavailable concentrations of the examined TEs, differences in their uptake by the investigated woody species and their stabilization in FA from the chronosequence lagoons at TENT A were species specific. All of the examined species exhibited low root-shoot transfer of Cr, while this was also determined for As in *T. tetrandra* and *A. fruticose*, which makes them good stabilizers of these Tes. However, due to high root-shoot transfer and toxic concentrations of As, B, and Zn in the leaves of planted *R. pseudoacacia* and spontaneously colonized *P. alba*, as well as Se in planted *T. tetrandra*, it can be concluded that they are not suitable for use in the phytostabilization of FA. On the other hand, *A. fruticosa* exhibited tolerance to B toxicity and the ability to keep concentrations of Zn, Cu, and Mn in leaves within a normal range. These results proved our hypothesis that this spontaneously colonized woody species, thanks to its high effectiveness in the stabilization of FA, is suitable for use in the sustainable revitalization of FA at the TENT A ash disposal site, as well as similar ash deposit sites worldwide. In addition, it has a whole range of advantages over the other examined species, such as its capacity for vegetative propagation, its extensive root system, its enriching FA with nitrogen, and its tolerance to the prevailing conditions at FA lagoons. Our findings also indicate that, thanks to a reduction in the negative effects that dispersal and leaching of TEs have on the environment and human health, *A. fruticosa* has great potential for the sustainable phytomanagement of FA disposal sites, which outweighs the environmental damage/costs arising from its invasive spreading. This should be confirmed by future research through a cost-benefit analysis of the impact of this species as an invasive and phytostabilizing species.

## Figures and Tables

**Table 1:** Selected physical and chemical properties of the surface layer (0–30 cm) of fly ash from the examined sites (L1 and L2).

		Sand	Silt + Clay									
Site		2.0–0.02 mm	<0.02 mm	pH_H2O_	pH_KCl_	EC	CEC	C	N	C/N	K_2_O	P_2_O_5_
		%	%			dS m^−1^	cmol kg^−1^	%	%		mg/100 g	mg/100 g
L1	M (SD)	83.33 (1.48) a	16.67 (1.48) b	7.95 (0.08) a	7.25 (0.05) a	0.27 (0.02) a	47.13 (3.90) b	1.85 (0.16) a	0.05 (0.00) b	37.0	44.40 (2.59) b	19.12 (1.35) a
L2	M (SD)	71.60 (2.51) b	28.40 (2.53) a	7.72 (0.02) b	6.74 (0.14) b	0.19 (0.01) b	67.02 (1.16) a	1.13 (0.23) b	0.11 (0.02) a	10.27	52.50 (0.41) a	10.01 (2.05) b

(ANOVA-Bonferroni); data represents the mean M with standard deviation (SD) of three replicates (*n* = 3); different letters in the same column indicate significant difference between sites at *p* < 0.05.

**Table 2:** Pseudototal concentration (C_PT_), bioavailable fraction (C_DTPA_), and mobility (AR) of trace elements in the surface layer (0–30 cm) of fly ash from the examined sites (L1 and L2).

	Site		As	B	Cr	Cu	Mn	Ni	Se	Zn
C_PT_ (mg kg^−1^)	**L1**	M (SD)	**21.62** (2.53) a	41.28 (4.33) a	**87.26** (9.79) a	49.51 (5.32) a	243.95 (11.0) a	73.97 (8.31) a	1.96 (0.11) a	49.95 (6.57) a
	**L2**	M (SD)	12.95 (0.53) b	22.37 (1.21) b	40.02 (4.84) b	36.12 (2.39) b	160.85 (9.01) b	86.27 (13.61) a	1.85 (0.51) a	19.55 (2.51) b
C_DTPA_ (mg kg^−1^)	**L1**	M (SD)	0.29 (0.01) b	1.71 (0.06) a	0.04 (0.00) a	0.86 (0.02) a	1.36 (0.04) a	0.54 (0.01) b	0.03 (0.01) a	0.82 (0.09) a
	**L2**	M (SD)	0.53 (0.02) a	0.31 (0.01) b	0.03 (0.00) b	0.48 (0.02) b	0.56 (0.03) b	1.02 (0.04) a	0.02 (0.01) a	0.42 (0.13) b
AR (%)	**L1**		1.36	4.14	0.04	1.72	0.56	0.73	1.64	1.65
	**L2**		4.12	1.40	0.06	1.34	0.35	1.18	1.25	2.14
Typical range in fly ash (mg kg^−1^) [11]			2–70	2–5000	3–900	10–2000	30–3000	10–3000	0.2–50	10–1000
Average range in soil (mg kg^−1^) [45]			4.4–8.4	22–40	47–51	13–23	270–525	13–26	0.25–0.34	45–60
Critical range in soil (mg kg^−1^) [77]			20–50	-	75–100	60–125	1500–3000	100	5–10	70–400

(ANOVA-Bonferroni); data represents the mean M with standard deviation (SD) of five replicates (*n* = 5); different letters in the same column for the same extraction method indicate significant difference between sites at *p* < 0.05; critical concentrations are in bold.

**Table 3:** Spearman’s correlation coefficient between bioavailable trace element concentrations in FA (C_DTPA_) and concentrations in roots (CRoot) and leaves (CLeaf) of the examined woody plant species.

Plants	Fly Ash (C_DTPA_)							
	As	B	Cr	Cu	Mn	Ni	Se	Zn
**CRoot**								
*T. tetrandra*	0.560	0.751 c	−0.834 b	0.990 a	0.954 a	0.297	−0.473	0.892 a
*A. fruticosa*	0.447	0.857 b	−0.235	−0.833 b	0.843 b	0.906 a	−0.594	−0.841 b
*P. alba*	−0.117	0.954 a	0.913 a	0.994 a	0.812 b	−0.887 a	0.010	0.774 b
*R. pseudoacacia*	−0.001	−0.306	0.905 a	−0.063	0.857 b	0.980 a	−0.229	0.716 c
**CLeaf**								
*T. tetrandra*	0.565	−0.993 a	−0.193	0.997 a	0.997 a	−0.884 a	−0.593	0.883 a
*A. fruticosa*	−0.143	0.998 a	−0.523	−0.984 a	0.983 a	0.970 a	−0.611	0.704 c
*P. alba*	0.877 a	0.998 a	0.955 a	0.690 c	0.986 a	−0.988 a	−0.157	−0.744 c
*R. pseudoacacia*	0.456	0.399	0.842 b	0.950 a	0.214	0.122	−0.661 c	0.774 b

Correlation is significant at a 0.001, b 0.01, c 0.05 level of significance.

**Table 4:** Spearman’s correlation coefficient between trace element concentrations in the roots (CRoot) and leaves (CLeaf) of the examined woody plant species.

CRoot	CLeaf							
	As	B	Cr	Cu	Mn	Ni	Se	Zn
*T. tetrandra*	0.638 c	−0.715 c	0.317	0.994 a	0.955 a	−0.082	0.103	0.997 a
*A fruticosa*	−0.365	0.868 b	0.108	0.837 b	0.830 b	0.917 a	0.949 a	−0.431
*P. alba*	−0.203	0.960 a	0.933 a	0.705 c	0.774 b	0.882 a	−0.167	−0.602
*R. pseudoacacia*	0.318	0.289	0.782 b	−0.058	0.170	0.095	0.593	0.875 a

Correlation is significant at a 0.001, b 0.01, c 0.05 level of significance.

## Data Availability

Not applicable.

## Supplementary Materials

The following supporting information can be downloaded at: https://www.mdpi.com/article/10.3390/plants11010110/s1, Table S1: Total concentrations of trace elements in the roots (CRoot) and leaves (CLeaf) of four woody plant species growing at the examined sites (L1 and L2). Table S2: The bioaccumilation (BAF) and translocation factor (TF) of trace elements in four woody plant species growing at the examined sites (L1 and L2).
