# Supplementary material for: An Assessment of the Phytoremediation Potential of Planted and Spontaneously Colonized Woody Plant Species on Chronosequence Fly Ash Disposal Sites in Serbia—Case Study
Source: Plants (Basel). 2021 Dec 30;11(1):110. doi: 10.3390/plants11010110 (PMC8747270; doi:10.3390/plants11010110)
Supplement: Supplementary file 1 [file plants-11-00110-s001.zip › plants-1498044-supplementary.pdf]

Table S1. Total concentrations of trace elements in the roots (CRoot) and leaves (CLeaf) of four woody plant species growing at the examined sites (L1 and L2)

Site	Species	As	B	Cr	Cu	Mn	Ni	Se	Zn
					**CRoot** mg kg^{-1} DW				
L1	*T. tetrandra*	4.75 (0.30) **b**	12.85 (0.77) **cd**	0.88 (0.07) **cd**	4.58 (0.11) **b**	8.13 (0.26) **cd**	3.68 (0.14) **ab**	1.95 (0.52) **a**	20.80 (0.39) **b**
	A. fruticosa	**5.27** (0.46) **ab**	17.32 (1.44) **b**	1.15 (0.05) **bcd**	3.62 (0.19) **d**	28.02 (1.77) **a**	3.37 (0.15) **b**	0.61 (0.13) **c**	13.05 (0.75) **d**
	P. alba	**5.28** (0.14) **ab**	23.28 (2.48) **a**	4.11 (0.53) **a**	6.58 (0.10) **a**	10.71 (1.72) **c**	3.81 (0.47) **ab**	1.03 (0.27) **bc**	24.92 (1.55) **a**
	R. pseudoacacia	4.72 (0.17) **b**	10.63 (0.61) **d**	1.05 (0.07) **bcd**	2.57 (0.14) **f**	5.69 (0.41) **de**	2.87 (0.08) **c**	0.53 (0.22) **c**	9.51 (1.42) **e**
L2	*T. tetrandra*	**5.04** (0.17) **ab**	10.96 (1.12) **cd**	1.27 (0.10) **bc**	2.92 (0.14) **e**	6.01 (0.46) **de**	3.77 (0.19) **ab**	2.07 (0.29) **a**	4.94 (0.42) **f**
	A. fruticosa	**5.64** (0.30) **a**	13.61 (1.00) **c**	1.15 (0.10) **bcd**	4.02 (0.10) **c**	21.85 (2.02) **b**	4.04 (0.14) **a**	1.67 (0.43) **ab**	17.35 (2.07) **c**
	P. alba	**5.26** (0.40) **ab**	11.78 (1.13) **cd**	1.32 (0.07) **b**	3.46 (0.14) **d**	7.20 (0.99) **d**	2.64 (0.18) **c**	1.01 (0.37) **bc**	21.20 (2.18) **b**
	R. pseudoacacia	4.77 (0.37) **b**	10.34 (0.32) **d**	0.80 (0.08) **d**	2.57 (0.18) **f**	4.57 (0.31) **e**	3.52 (0.06) **b**	0.92 (0.26) **c**	6.42 (0.34) **f**
					**CLeaf** mg kg^{-1} DW				
L1	*T. tetrandra*	3.17 (0.07) **d**	41.31 (1.02) **g**	0.9 (0.10) **b**	17.91 (0.22) **a**	46.16 (0.50) **a**	3.1 (0.17) **d**	**10.83** (0.81) **b**	48.48 (0.68) **c**
	A. fruticosa	4.12 (0.69) **bc**	**115.86** (0.83) **c**	0.4 7(0.06) **e**	7.34 (0.30) **d**	19.37 (0.21) **d**	3.40 (0.10) **d**	0.90 (0.10) **ef**	22.05 (0.36) **e**
	P. alba	4.46 (0.39) **b**	**401.33** (4.00) **a**	1.24 (0.00) **a**	7.67 (0.32) **d**	22.71 (0.96) **c**	6.38 (0.12) **a**	1.62 (0.46) **de**	**100.74** (3.42) **b**
	R. pseudoacacia	**5.55** (0.20) **a**	**170.00** (7.54) **b**	0.62 (0.00) **d**	5.85 (0.17) **e**	20.34 (0.56) **d**	3.19 (0.07) **d**	0.54 (0.08) **f**	19.66 (0.27) **ef**
L2	*T. tetrandra*	3.51 (0.34) **cd**	**58.84** (1.21) **f**	1.00 (0.00) **b**	11.32 (0.31) **b**	24.50 (0.46) **b**	2.75 (0.01) **e**	**16.42** (0.45) **a**	34.00 (0.63) **d**
	A. fruticosa	3.98 (0.27) **bcd**	**73.46** (1.10) **e**	0.50 (0.00) **de**	10.33 (0.25) **c**	15.48 (0.46) **e**	4.20 (0.11) **c**	2.58 (0.50) **c**	21.74 (0.21) **e**
	P. alba	**5.61** (0.32) **a**	**95.17** (0.76) **d**	0.77 (0.10) **c**	7.24 (0.13) **d**	12.88 (0.25) **f**	5.20 (0.17) **b**	2.45 (0.34) **cd**	**111.21** (0.64) **a**
	R. pseudoacacia	**5.97** (0.56) **a**	**165.44** (2.95) **b**	0.45 (0.07) **e**	4.90 (0.24) **f**	20.14 (0.42) **d**	3.20 (0.19) **d**	2.18 (0.23) **cd**	17.88 (0.36) **f**
Deficient concentration [45]		-	5-30	-	2-5	10-30	-	-	10-20
Normal concentration [45]		1-1.7	10-100	0.1-0.5	5-30	30-300	0.1-5	0.01-2	27-150
Normal concentration *[77]*									10-300
Excessive or toxic concentration [45]		**5-20**	**50-200**	**5-30**	**20-100**	**400-1000**	**10-100**	**5-30**	**100-400**
Excessive or toxic concentration *[77]*									**>400**

(ANOVA- Bonferroni); Data represents the mean M with standard deviation (SD) of five replicates (n=5); DW – dry weight, Different letters in the same column indicate significant difference at P <0.05; Toxic concentrations are in bold

Table S2. The bioaccumilation (BAF) and translocation factor (TF) of trace elements in four woody plant species growing at the examined sites (L1 and L2)

			As	B	Cr	Cu	Mn	Ni	Se	Zn
**BAF**	**L1**	*T. tetrandra*	16.16	7.51	23.16	5.31	6.00	6.84	60.94	25.24
		A. fruticosa	17.92	10.13	30.26	4.20	20.68	6.26	19.06	15.84
		P. alba	17.90	13.61	108.16	7.63	7.90	7.08	32.19	30.24
		R. pseudoacacia	16.05	6.22	27.63	2.98	4.20	5.33	16.56	11.54
	**L2**	*T. tetrandra*	9.47	35.13	42.33	6.02	10.77	3.69	90.00	11.79
		A. fruticosa	10.60	43.62	38.33	8.29	39.16	3.32	72.61	41.41
		P. alba	9.89	37.76	44.00	7.13	12.90	2.58	43.91	50.60
		R. pseudoacacia	8.97	33.14	26.67	5.30	8.19	3.44	40.00	15.32
**TF**	**L1**	*T. tetrandra*	0.67	3.21	1.10	3.91	5.68	0.85	5.55	2.33
		A. fruticosa	0.78	6.69	0.41	2.03	0.69	1.01	1.48	1.69
		P. alba	0.84	17.24	0.30	1.16	2.12	1.67	1.57	4.04
		R. pseudoacacia	1.18	15.99	0.59	2.28	3.57	1.11	1.02	2.07
	**L2**	*T. tetrandra*	0.70	5.37	0.79	3.88	4.08	0.73	7.93	6.88
		A. fruticosa	0.70	5.40	0.43	2.57	0.71	1.04	1.54	1.25
		P. alba	1.07	8.08	0.58	2.09	1.79	1.97	2.42	5.24
		R. pseudoacacia	1.25	16.00	0.56	1.91	4.41	0.91	2.37	2.78
